# Inhibitory Activity of Allergic Contact Dermatitis and Atopic Dermatitis-Like Skin in BALB/c Mouse through Oral Administration of Fermented Barks of *Alnus sibirica*

**DOI:** 10.3390/molecules23020450

**Published:** 2018-02-18

**Authors:** Jun Yin, Seong Hye Yoon, Hye Shin Ahn, Min Won Lee

**Affiliations:** Laboratory of Pharmacognosy and Natural Product based Medicine, College of Pharmacy, Chung-Ang University, Seoul 156-756, Korea; yinjun89@naver.com (J.Y.); lily6810@naver.com (S.H.Y.); hyaeshin@naver.com (H.S.A.)

**Keywords:** *Alnus sibirica* Fisch. ex Turcz., fermentation, diarylheptanoid, antioxidant, anti-inflammatory, anti-allergic contact dermatitis, anti-atopic dermatitis

## Abstract

Phytochemical isolation of fermented *Alnus sibirica* (FAS) which was produced by using *Lactobacillus plantarum* subsp. *argentoratensis*, exhibited multiple and different composition compared with the original plant. Anti-allergic contact dermatitis (anti-ACD)/anti-atopic dermatitis (anti-AD) activities (visual observation and regulation of Th_1_/Th_2_ cytokines and IgE in blood) of FAS and the barks of *Alnus sibirica* extract (AS) and the two diarylheptanoids, hirsutenone (**1**) and muricarpon B (**2**), which are major components of FAS, were measured in vitro and in vivo. FAS, AS and the two compounds showed potent anti-oxidative, anti-inflammatory, anti-ACD and anti-AD activity. In particular, FAS showed more potent biological activity than AS. Thus, fermentation might be a prominent way to enhance the biological activity compared with the original plant. In addition, compounds (**1**) and (**2**) might be developed as functional materials or herbal medicines for ACD and AD.

## 1. Introduction

Contact dermatitis (CD) is an inflammatory skin reactions induced by exposure to external agents. The type of CD including irritants contact dermatitis (ICD) and allergic contact dermatitis (ACD) result from tissue damage by contact with irritant and allergen. Atopic dermatitis (AD) is a chronic inflammatory skin disease that can result in red, swollen, cracked skin that usually begins from childhood. AD affects 15–20% of children [1] and 1–3% adults worldwide [2]. The cause of AD is unknown, although there is some evidence of genetic, environmental and immunologic contributors [3], and atopic reactions are generally considered the cause by localized hypersensitivity reaction to an allergen. It is reported that AD is frequently associated with increased IgE and Th_2_ cytokine at the early stage including interleukin (IL)-4, IL-5, IL-10 and IL-13 and the tumor necrosis factor (TNF)-α [4,5,6]. Because of the decreased Th_1_ cytokines, the imbalance of Th_1_ and Th_2_ cytokines cause AD. However, in the later stage of AD, interferon (IFN)-γ was higher in the late skin lesions of human patients and mice [7,8,9]. Topical steroids, such as triamcinolone or clobetasol are successfully treat AD [10]. However, they can also increase the risk of skin cancer or lymphoma [11]. Thus, natural plants, which are safe and have fewer side effects, have been recommended [12].

The fermentation of traditional medicine has a long history in China, Korea, and India among other countries, and today, fermented traditional medicines are widely applied for preventing and treating many diseases. [13,14,15] Generally, fermentation is a metabolic process that converts sugars to acids, gases, or alcohol and degrades the organic components through oxidation–reduction. Herbal medicines have been fermented into beneficial micro-organisms such as yeast and lactobacillus, which could develop the bioactivity of natural plants and reduce side effects. [15]

Alnus species have been used for galactogogues, cathartics, hemostatics, emetics, febrifuges, skin tonic and parasiticides [16]. The bark of *Alnus sibirica*, which is distributed in Korea, Japan, Northeast China and Russia, has been reported for use as antipyretic, expectorant, antiasthmatic and a health tea for alcoholism [17]. Previous works have studied the phenolic components of various tannins, flavonoids, diarylheptanoids and triterpenoids that were isolated from the *Alnus* species [18]. In addition, *Alnus japonica* and diarylheptanoids, which are major compounds in AS, have been attempted for overcoming diseases including AD [19,20,21].

**Figure 1:**
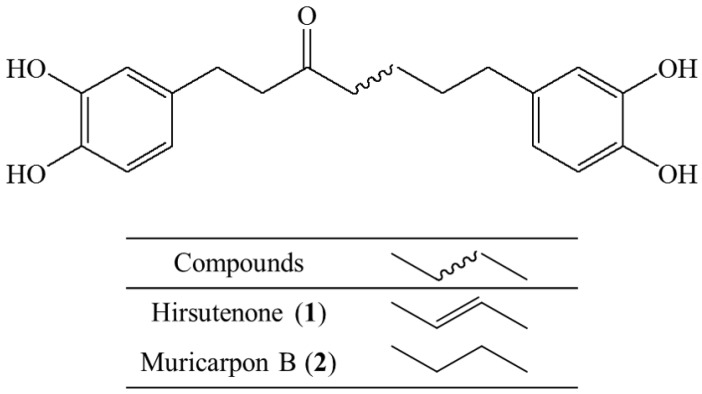
Structures of hirsutenone (**1**) and muricarpon B (**2**).

The present study investigated the bioactivity of fermented AS (FAS) and two isolated compounds, hirsutenone (**1**) and muricarpon B (**2**), which are main components of FAS, (Figure 1) in anti-oxidative, anti-inflammatory and anti-ACD and anti-AD experiments in vitro and in vivo.

## 2. Results and Discussions

### 2.1. Anti-Oxidative Activity

From, the DPPH results, all extracts and compounds showed potent dose-dependent radical scavenging activities. In particular, compared with AS (IC_50_: 31.41 μg/mL), FAS (IC_50_: 24.34 μg/mL) showed more potent radical scavenging activities. In addition, **1** and **2** (IC_50_: 14.08 μM, 24.08 μM) showed more potent radical scavenging activities than did the positive control, *l*-Ascorbic acid (IC_50_: 24.81 μM; Table 1). Additionally, the results for NBT superoxide radical scavenging activity were similar to the DPPH results; FAS (IC_50_: 1.11 μg/mL) showed more potent superoxide radical scavenging activities than did AS (IC_50_: 6.57 μg/mL). 

Oxidative stress is an imbalance between the production of reactive oxygen species (ROS) and antioxidant defense, and excess ROS could damage cellular protein, DNA and lipids, resulting in various diseases such as Parkinson’s disease, Alzheimer’s disease, cancer and atopic disease [22,23,24,25]. Thus, anti-oxidative activity may be beneficial in the ACD and AD. The 1,1-diphenyl-2-picrylhydrazyl (DPPH; Sigma-Aldrich, St. Louis, MO, USA) radical scavenging activity and nitro blue tetrazolium chloride (NBT) superoxide radical scavenging activity were measured. All extracts and compounds showed potent dose-dependent anti-oxidative activities. In particular, FAS (extract level) and **1** (compound level) showed more potent anti-oxidative activities, in both DPPH radical scavenging activity and NBT superoxide radical scavenging activity assays. Moreover, **1**, a potent antioxidant agent [26], showed more potent anti-oxidative activity than **2** (Table 1).

### 2.2. Anti-Inflammatory Activity

**Figure 2:**
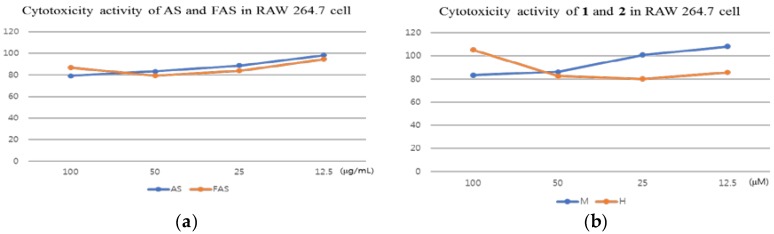
Cytotoxicity activity in RAW 264.7 cell. (**a**): cytotoxicity activity of AS and FAS in RAW 264.7 cell; (**b**): cytotoxicity activity of **1** and **2** in RAW 264.7 cell. X axis: concentration of dose, Y axis: viability activity (%). The results were expressed as mean ± S.D. of triplicated experiments. (*n* = 3)

Before the measuring of anti-inflammatory activity experiments, the cytotoxicity activity of AS, FAS, **1** and **2** were studied on the macrophage cell line (RAW 264.7 cell line). The results showed that all the samples have no cytotoxicity activities on RAW 264.7 cells. (Figure 2)

FAS (IC_50_: 13.00 μg/mL) showed more potent inhibition of NO production than did AS (IC_50_: 19.31 μg/mL), and **1** and **2** (IC_50_: 14.10 μM, 22.66 μM) showed more potent inhibition activity than did the positive control, *N*^G^-monomethyl-l-arginine (L-NMMA; IC_50_: 37.05 μM; Table 2).

Nitric oxide (NO), which is a free radical, is an important cellular signaling molecule that is involved in many physiological and pathological processes in humans [27]. NO in large quantities is generated by inducible NO synthases and is reported to have an important role in inflammation and immune responses [28]. Thus, the inhibitory activity of NO production on FAS, AS, **1**, and **2** in the LPS-stimulated RAW264.7 cells was studied. FAS showed more potent anti-inflammatory activity than did AS, and both **1** and **2** showed more potent anti-inflammatory activity than did the positive control (Table 2), showing that **1** and **2** have excellent anti-inflammatory activity.

### 2.3. Anti-ACD and Anti-AD Activity in Vitro

**Figure 3:**
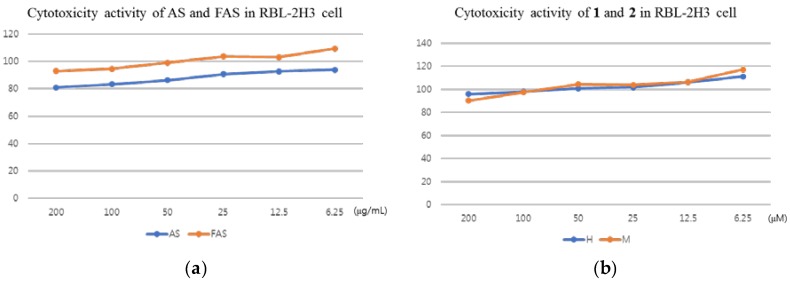
Cytotoxicity activity in RAW 264.7 cell. (**a**): Cytotoxicity activity of AS and FAS in RBL-2H3 cell; (**b**): cytotoxicity activity of **1** and **2** in RBL-2H3 cell. X axis: concentration of dose, Y axis: viability activity (%). The results were expressed as mean ± S.D. of triplicated experiments (*n* = 3).

Before the measuring of cytokine regulation in vitro, the cytotoxicity activities of AS, FAS, **1** and **2** were also studied on a basophils cell line (RBL-2H3 cell line). The results showed that all the samples have no cytotoxicity activities on RBL-2H3 cells. (Figure 3).

**Figure 4:**
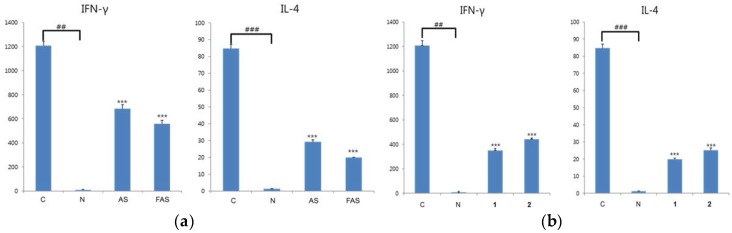
Anti-ACD/AD experiment in vitro. (**a**): Inhibitory activities of IFN-γ and Th_2_ on extract level in RAW 264.7 cells; (**b**): Inhibitory activities of Th_1_ and Th_2_ on compound level in RAW 264.7 cells. The results were expressed as mean ± S.D. of triplicated experiments. (*n* = 3). C: Negative control group; N: Normal control group. Y axis: concentration of cytokine (pg/mL) *** *p* < 0.001, comparing with negative control group; ^###^ *p* < 0.001, ^##^ *p* < 0.01, comparing with normal control group.

The regulation effects of IFN-γ and IL-4 cytokines on AS, FAS, **1** and **2** were measured. All samples showed down-regulation effects on IFN-γ and IL-4 cytokines. In particular, FAS showed more potent inhibition of IFN-γ and IL-4 cytokines than did AS in vitro by ELISA in RAW 264.7 cell lines (Figure 4a). In addition, **2** showed more potent inhibition of the two cytokines than **1** (Figure 4b).

**Figure 5:**
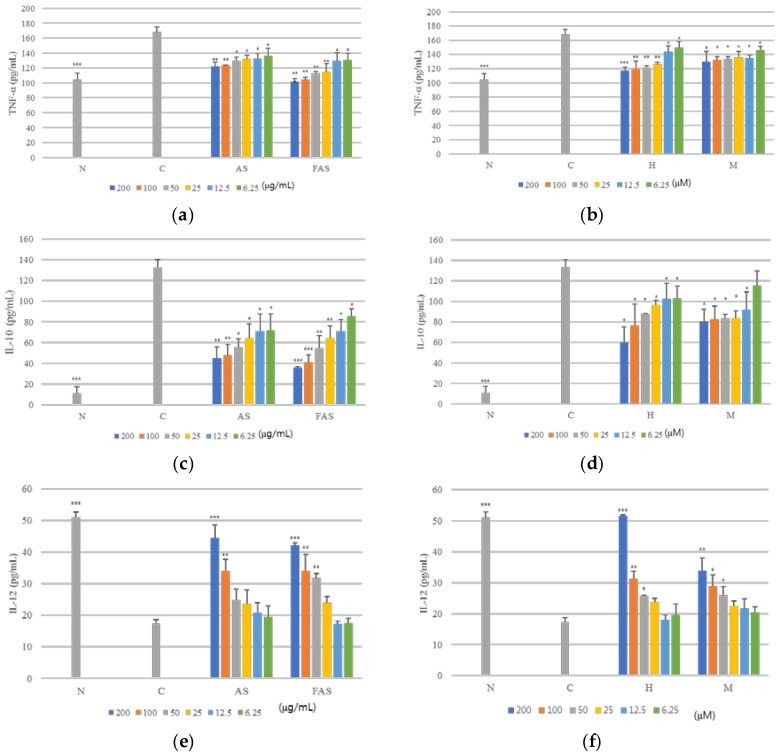
Anti-ACD/AD experiment in vitro. (**a**): regulation of TNF-α on extract level in RBL-2H3 basophils cell lines; (**b**): regulation of TNF-α on compound level in RBL-2H3 basophils cell lines; (**c**): regulation of IL-10 on extract level in RBL-2H3 cells; (**d**): regulation of IL-10 on compound level in RBL-2H3 cells; (**e**): regulation of IL-12 on extract level in RBL-2H3 cells; (**f**): regulation of IL-12 on compound level in RBL-2H3 cells. The results were expressed as mean ± S.D. of triplicated experiments. (*n* = 3). C: Negative control group; N: Normal control group. X axis: concentration of dose, Y axis: concentration of cytokine (pg/mL) *** *p* < 0.001, ** *p* < 0.01, * *p* < 0.05, comparing with negative control group.

Moreover, the regulation of other cytokines, like IL-10, IL-12 and TNF-α, were also measured. FAS showed more potent inhibition of TNF-α and IL-10 cytokines than AS in vitro by ELISA in the RBL-2H3 basophil cell line, (Figure 5a,c) and **1** also showed more potent inhibition of TNF-α and IL-10 cytokines than **2** in RBL-2H3 cells. (Figure 5b,d). However, all the samples (AS, FAS and compounds) increased the levels of IL-12 in the RBL-2H3 basophil cell line. (Figure 5e,f).

ACD, including AD, patients tend to have great hyper production of IgE, which is a result of the imbalance of T helper (Th_1_ and Th_2_) cells. Th_2_ cytokines, particularly IL-4 and IL-13, are potent stimulators of IgE synthesis [29,30], and other cytokines, such as IL-5 and IL-10, facilitate IgE production [29,31,32]. The down-regulation of Th2 cytokines may decrease production of IgE. In contrast, Th_1_ cytokines have negative effects on IgE synthesis, such as IFN-γ and IL-12 [29,30,33]. However, in the later stage of AD, the high concentration of IFN-γ existed in the AD later stage cases [7,8,9]. TNF-α, a proinflammatory cytokine, has been shown to produce some effects that are associated with infection, inflammation and immunoregulation [34,35,36,37] and it is reported that there is an increased TNF-α concentration in ACD/AD [38,39]. Our results showed that AS, FAS, **1** and **2** inhibited IL-4, IL-10, TNF-α and IFN-γ potently, but up-regulated the IL-12 cytokine. Although the samples decreased the IFN-γ cytokine, Th_2_ cytokines also were inhibited, this may be due to the increased IL-12 cytokine which is a cytokine involved in the production of IFN-γ [40,41].

### 2.4. Anti-ACD/Anti-AD Evaluation on BALB/c Mice

The regulation activity of FAS, AS, **1**, and **2** on Th_1_ (IFN-γ) and Th_2_ (IL-4) cytokines, which are increased in the early and late stages of ACD/AD, were evaluated [42,43]. In addition, we used the scoring atopic dermatitis (SCORAD) index and IgE levels in blood to measure the skin serious index. The SCORAD index is a clinical tool for assessing the severity of atopic dermatitis (AD) that was developed by the European Task Force on AD in 1993 [44]. The SCORAD index measures area, intensity and subjective symptoms. Intensity refers to dryness, redness, swelling, scabs, scratch marks and thickening of the skin, and meanwhile, IgE is an immunoglobulin isotype that is increased in ACD/AD. The study used 2,4-dinitrochlorobenzene (DNCB)-induced BALB/c mice as ACD/AD models and treated them with 100 mg/kg of extract (FAS and AS) and 100 μM/kg of compounds **1** and **2** by oral administration.

**Figure 6:**
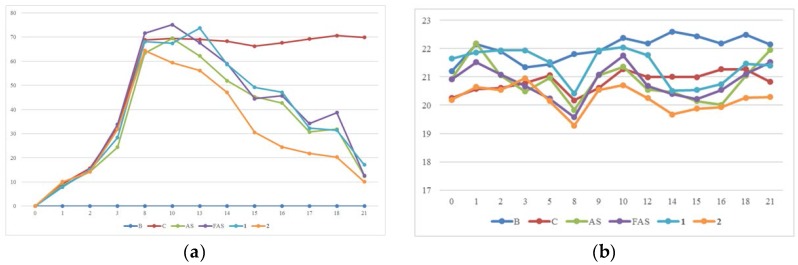
The condition of ACD/AD model animals after induced ACD/AD and treatment by experimental doses. (**a**): SCORAD indices for AS, FAS, **1** and **2**. X axis: Day of experiment, Y axis: The score of SCORAD; (**b**): Body weights of the experiment animals. X axis: Day of experiment, Y axis: Body weight of Balb/C mice (g). The results were expressed as mean ± S.D. of triplicate experiments. (*n* = 5). C: Negative control group; B: Blank normal control group.

As noted above, the SCORAD index measures area, intensity and subjective symptoms. Intensity measures dryness, redness, swelling, scabs, scratch marks and thickening of the skin, and the symptoms are assessed as none (0), mild (1), moderate (2), or severe (3); the subjective symptoms include itching and sleeplessness, and each is rated on a scale of 0–10. The SCORAD index is calculated using the following formula: A/5 + 7B/2 + C. The lowest SCORAD index of the compound **2** experiment group showed that **2** was the best agent for treating ACD/AD; even though AS, FAS and **1** also showed potent anti-ACD/AD effects (Figure 6a), and the body weights of the experimental animals showed no significant differences indicating that AS, FAS, **1** and **2** had no toxicity.

**Figure 7:**
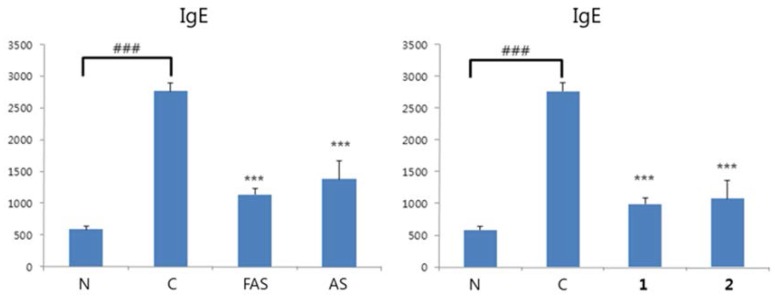

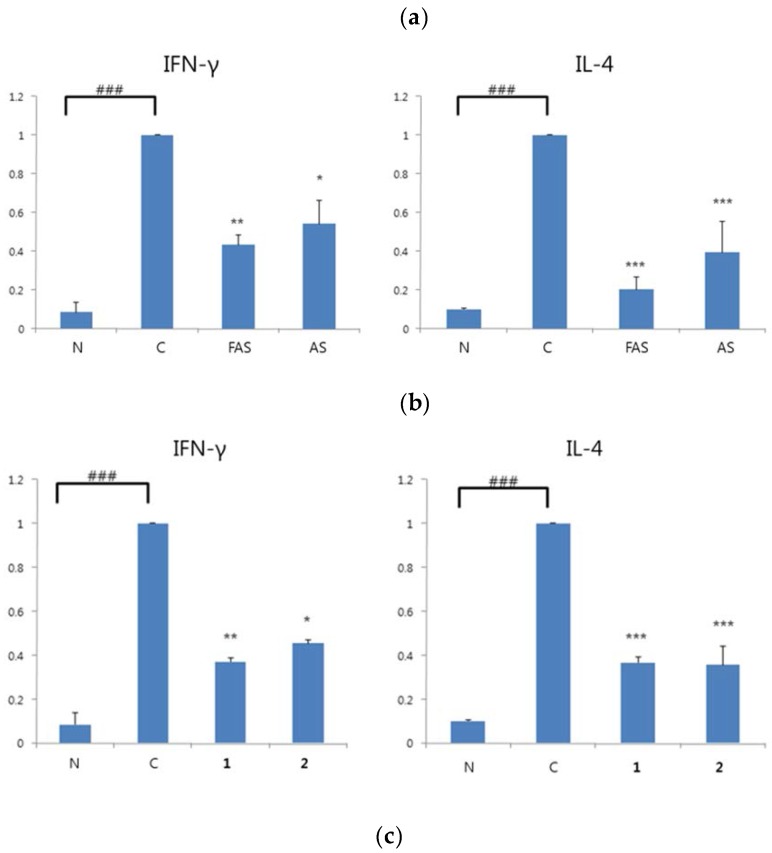
Anti-ACD/AD experiment in vivo. (**a**): Inhibitory activities of IgE levels in blood on extract level and compound level. Y axis: Concentration of cytokine (pg/mL); (**b**): Inhibitory activities of Th_1_ and Th_2_ on extract level in spleen. Y axis: Relative quantification of cytokine; (**c**): Inhibitory activities of Th_1_ and Th_2_ on compound levels in spleen. Y axis: Relative quantification of cytokine. The results were expressed as mean ± S.D. of triplicate experiments. (*n* = 5). C: Negative control group; N: Normal control group. *** *p* < 0.001, ** *p* < 0.01, * *p* < 0.05, comparing with negative control group; ### *p* < 0.001, comparing with normal control group.

The resulting SCORAD index showed that all samples including FAS, AS, **1** and **2** are good agents for treating AD/ACD (Figure 6a). Meanwhile, the body weights of the experimental animals showed no significant differences (Figure 6b). In the ACD/AD model, DNCB-induced BALB/c mouse, high levels of IgE, IL-4 and IFN-γ cytokines (BD Bioscience, San Diego, CA, USA) were detected (Figure 7), and all of the dose-treatment groups showed potent inhibition of these cytokines.

## 3. Materials and Methods 

### 3.1. Plant Material 

The bark of AS was collected from Mt. Kuksa, Seoul, Republic of Korea, in January 2015. The identification of the material was certified by Prof. Minwon Lee (Pharmacognosy Lab, College of Pharmacy, Chung-Ang University, Seoul, Korea), and a voucher specimen was deposited at the pharmacy department’s herbarium (20150816AS).

### 3.2. Extraction, Fermentation and Isolation

The bark of AS was extracted with 80% prethanol A at room temperature and, then, the prethanol A was removed by vacuuming the AS extract. The AS was fermented by using Lactobacillus plantarum with MRS broth for 7 days at room temperature. The FAS was subjected to Sephadex^®^ LH-20 (GE Healthcare, Uppsala, Sweden) column chromatography and MCI gel (SUPELCO, Bellefonte, PA, USA) column yielded **1** and **2**. They were compared with standard co-TLC.

### 3.3. Measuring DPPH Radical Scavenging Activity

Each 20 μL of sample was added to 180 μL of 0.1 mM DPPH (Sigma-Aldrich, St. Louis, MO, USA) in absolute ethanol. After the samples were mixed gently and left to stand for 30 min, the optical density (*O.D.*) of the samples was measured at 518 nm using an ELISA reader (TECAN, Salzburg, Austria). The free radical scavenging activity was calculated as inhibition rate (%) = 100 − (sample *O.D.*/control *O.D.*) × 100; IC_50_ is the concentration of 50% DPPH free radicals scavenge activity. *l*-ascorbic acid is the positive control.

### 3.4. Measuring NBT Superoxide Radical Scavenging Activity

A reaction mixture with a final volume of 632 μL/Eppendorf was prepared with 50 mM phosphate buffer (pH 7.5) containing EDTA (0.05 mM), hypoxanthine (0.2 mM), 63 μL NBT (1 mM; Sigma-Aldrich, St. Louis, MO, USA), 63 μL of aqueous or ethanolic extract (distilled water for the control), and 63 μL of xantine oxidase (1.2 U/μL; Sigma); the xanthine oxidase was added last. For each sample, a blank was carried out. The subsequent rate of NBT reduction was determined based on sequential spectrophotometric determinations of absorbance at 590 nm. The solutions were prepared daily and kept from light. The results are expressed as the percentage inhibition of the NBT reduction with respect to the reaction mixture without the sample (buffer only). Superoxide anion scavenging activity was calculated as ((1−(sample *O.D.*− blank *O.D.)*/(control *O.D*. − blank *O.D*.)) × 100); IC_50_ was defined as the concentrations at which 50% of NBT/superoxide anions were scavenged. Allopurinol was used as the positive control.

### 3.5. Cell Culture

RAW 246.7 cells and RBL-2H3 cells were purchased from the Korean Cell Line Bank. The cells were grown at 37 °C in a humidified atmosphere (5% CO_2_) in Dulbeccos’s Modified Eagles’s Medium (DMEM, Sigma-Aldrich containing, 10% fetal bovine serum (FBS) and 100 IU/mL penicillin G (Gibco BRL, Grand Island, NY, USA)).

### 3.6. Viability Assay

Approximately 10^5^/well of RAW264.7 or RBL-2H3 cells were seeded in wells of a 96-well plate and incubated for 4 h in 5% CO_2_ at 37 °C. The medium was replaced with phosphate-buffered saline (PBS) containing 1 mg/mL of 3-(4,5-dimethylthiazol-2-yl)-2,5-diphenyltetrazolium bromide (MTT), and incubated for 4 h. The supernatant was removed and the MTT-formazan was dissolved in 100 μL dimethylsulfoxide. The extent of the reduction of MTT to formazan within the cells was measured at 540 nm with microplate reader (TECAN, Salzburg, Austria). The cell viability was calculated as sample O.D./blank *O.D*.× 100 (%).

### 3.7. Measuring NO Production Inhibition

RAW 264.7 cells were cultured in 96-well plates and incubated for 3 h at 37 °C in a humidified atmosphere of 5% CO_2_. Then the cells were treated with 1 mg/mL lipopolysaccharide (LPS; Sigma-Aldrich) and incubated for 24 h. The NO content was determined by Griess assay. After aspiration of the supernatant, the 100 μL of Griess reagent (0.1% naphthylethylenediamine and 1% sulfanilamide in 5% H_3_PO_4_ solution; Sigma-Aldrich) was added to each well, then, 100 μL of DMSO were added. The content of NO was then measured at 540 nm with an ELISA reader (TECAN, Salzburg, Austria). NO inhibitory activity was calculated as 100 − (sample *O.D.* − blank *O.D.*)/(negative control *O.D.* − blank *O.D.*) × 100 (%); IC_50_ is the concentration of 50% inhibitory activity of NO production. *N*^G^-monomethyl-l-arginine (L-NMMA) was used as the positive control.

### 3.8. Measuring Cytokine Production Inhibition

The concentrations of cytokines (IFN-γ, IL-4, and IgE [BD Bioscience]; IL-10, IL-12p70, TNF-α (AFfrontier)) in culture supernatants were measured by ELISA. Cytokine content was quantified by measuring the absorbance at 405 nm with an ELISA reader (TECAN), and the amounts of cytokines produced were calculated using a standard calibration curve. After the cells were exposed by LPS, the IFN-γ, IL-4 cytokine levels were measured for the regulation effects of AS, FAS, **1**, and **2** at 100 μg/mL and 100 μM, and IL-10, IL-12p70 and TNF-α cytokine levels were measured for the regulation effect of AS, FAS, **1** and **2** at 200~6.25 μg/mL and 200~6.25 μM.

### 3.9. Animal Experiment Model

Male BALB/c mice (7 weeks old) were adapted to laboratory conditions (temperature: 20 ± 2°C, relative humidity: 50%, light/dark cycle: 12 h) for 1 week; The animal experiments were approved by the Chung-Ang University Institute for Animal Research. Thirty BALB/c mice were randomly divided into six groups: the normal group; the negative control group; the AS treatment group; the FAS treatment group; the **1** treatment Group; and the M treatment group. 2,4-dinitrochlorobenzene (DNCB, Sigma-Aldrich) dissolved in acetone was used to induce dermatitis in the BALB/c mice except for in the normal group. The outer ears were sensitized with 20 μL of 1% DNCB daily for 7 days. After the first challenge, 20 μL of 0.5% DNCB was repeatedly applied to the outer ears for an additional 14 days at 2-day intervals. At the second challenge, FAS and AS (100 mg/kg) and **1** and **2** (100 μM/kg) were administered orally. After 14 days, all the mice were sacrificed. The animal studies have been reviewed and approved by the Institutional Animal Care and Use Committee (IACUC) at CHUNG-ANG UNIVERSITY. (2016-00037)

### 3.10. Preparing the Spleen Microsomes

Mice spleens were prepared from the experimental mice. After the spleens were washed in phosphate-buffered saline (PBS), they were mashed and centrifuged at 400G (gravitation) for 2 min. The supernatant was removed and mixed with 1X RBC lysis buffer at room temperature for 5 min. The mixtures were washed by PBS and centrifuged at 400 G for 2 min. Then the supernatant was removed and mixed with PBS and filtered using an 800 mesh filter. After centrifugation with 400 G for 2 min, the Th_1_ and Th_2_ cytokines in the spleen cells were measured using real-time polymerase chain reaction (PCR). 

Mice spleens were prepared from the experimental mice. After the spleens were washed in phosphate-buffered saline (PBS), they were mashed and centrifuged at 400G (gravitation) for 2 min. The supernatant was removed and mixed with 1X RBC lysis buffer at room temperature for 5 min. The mixtures were washed by PBS and centrifuged at 400 G for 2 min. Then the supernatant was removed and mixed with PBS and filtered using an 800 mesh filter. After centrifugation with 400 G for 2 min, the Th_1_ and Th_2_ cytokines in the spleen cells were measured using real-time polymerase chain reaction (PCR). 

### 3.11. Reverse Transcription RT-PCR

Total RNA was isolated from the spleen cells using TRIzol reagent (Invitrogen, Waltham, MA, USA), and spleen microsomes were added to 1 ml of TRIzol reagent in a cultured dish. After 5 min at room temperature, 0.2 mL of chloroform per 1 mL of TRIzol reagent was added, and the tubes were shaken vigorously by hand for 15 seconds. The mixtures were centrifuged at 13,500 rpm and 4 °C for 15 min, 500 μL of the upper aqueous phase was transferred to a fresh tube, and iso-propanol (500 μL) was added. 

After the mixtures were incubated at room temperature for 10 min, they were centrifuged at 13,500 rpm and 4 °C for 10 min. The supernatant was removed, washed with 700 μL of 70% ethanol, and centrifuged at 11,500 rpm and 4 °C for 10 min, the RNA pellet was briefly dried. The purified RNA was dissolved in diethyl pyrocarbonate-distilled water (DEPC-DW); 1 μg RNA was reverse transcripted at 42 °C for 1 h in a reaction containing reverse transcriptase (TaKaRa, Shiga, Japan), with 10× RT buffer, 10 mM dNTP (dNTP mix), oligo dT primer and RNase inhibitor. The 1 μL cDNA sample was amplified with 10 μL 2× ReddyMix PCR Master Mix, 2 μL 10 pM primer (1 μL 10 pM upstream primer, 1 μL 10 pM downstream primer), 7 μL DEPC-DW, and Universal SYBR Green Supermix using real-time PCR (BioRAD, Hercules, CA, USA). PCR primers were chemically synthesized using a DNA synthesizer (Bioneer, Daejeon, Korea), and the amplification conditions were: denaturation at 95 °C for 5 min for the first cycle and for 30 s starting from the second cycle, annealing at 51 °C for 30 s, 72 °C for 30 s. Final extension was performed at 72 °C for 10 min. The primers were β-actin (187 bp): 5′-TACAGCTTCACCACCACAGC-3′ (sense), 5′-AAGGAAGGCTGGAAAAGAGC-3′ (anti-sense)/ IL-4 (252 bp): 5′-ATATCCACGGATGCGACAAA-3′ (sense), 5′-AAGCCCGAAAGAGTCTCTGC-3′ (sense)/IFN-γ (193 bp): 5′-TGAAAATCCTGCAGAGCCAG-3′(sense), 5′-TGGACCTGTGGGTTGTTGAC-3′ (anti-sense).

### 3.12. Statistical Analysis

Values were analyzed by one-way analysis of variance (ANOVA) followed by Student-Newman-Keuls (S-N-K) test and one to one confrontation test that figure out *t*-value, *p*-value with Statistical Package for the Social Sciences (SPSS) software pack (v.18.0, IBM, Armonk, New York, NY, USA).

## 4. Conclusions

FAS was produced by fermenting AS with lactobacillus, and the phytochemical isolation of FAS yielded **1** as a main component and **2**, which was newly produced. The anti-ACD/AD activities of AS, FAS, **1** and **2** were tested in vitro, and FAS showed more potent anti-ACD/AD activity than AS. Moreover, **2**, the newly isolated FAS, showed more potent anti-ACD/AD activity than **1**, which had been reported to be a potent anti-ACD/AD agent [20]. These results suggest that FAS, AS, **1** and **2** are prominent anti-ACD/AD agents. 

## Figures and Tables

**Table 1:** IC_50_ values for FAS, AS, H, and M against DPPH and NBT radical scavenging activity.

Samples	DPPH Radical Scavenging Activity		NBT Radical Scavenging Activity	
	IC_50_ (μg/mL)	IC_50_ (μM)	IC_50_ (μg/mL)	IC_50_ (μM)
FAS	24.34 ± 0.9		1.11 ± 0.36	
AS	31.41 ± 1.14		6.57 ± 0.44	
**1**		14.08 ± 0.13		17.56 ± 0.29
**2**		24.08 ± 1.08		30.16 ± 2.85
l-Ascorbic acid	14.68 ± 0.36	24.81 ± 0.59		
Allopurinol			0.95 ± 0.2	7.99 ± 0.66

Values are presented as mean ± SD (*n* = 3).

**Table 2:** IC_50_ values for FAS, AS, H, and M against inhibiting NO production on LPS-stimulated RAW264.7 cells.

Samples	Inhibition of NO Production	
	IC_50_ (μg/mL)	IC_50_ (μM)
FAS	13.00 ± 0.38	
AS	19.31 ± 1.22	
**1**		14.10 ± 0.20
**2**		22.66 ± 1.18
L-NMMA	2.22 ± 0.11	37.05 ± 1.21

Values are presented as mean ± SD (*n* = 3).
